# Application of an Automatic Medical Information System to Implement Bundle Care for the Prevention of Ventilator-Associated Pneumonia

**DOI:** 10.3390/ijerph182111128

**Published:** 2021-10-22

**Authors:** Hung-Hui Lee, Li-Ying Lin, Hsiu-Fen Yang, Yu-Yi Tang, Pei-Hern Wang

**Affiliations:** 1 Department of Nursing, Kaohsiung Veterans General Hospital, Kaohsiung City 813414, Taiwan; hhlee@vghks.gov.tw (H.-H.L.); sfyoung@vghks.gov.tw (H.-F.Y.); uytang@vghks.gov.tw (Y.-Y.T.); phwang@vghks.gov.tw (P.-H.W.); 2 Department of Nursing, Meiho University, Neipu, Pingtung 91202, Taiwan

**Keywords:** ventilator-associated pneumonia, bundle, intensive care, information system

## Abstract

Ventilator-associated pneumonia is a common hospital-acquired infection. It causes patients to stay longer in the hospital and increases medical costs. This study explores the effect of applying an automatic medical information system to implement five-item prevention care bundles on the prevention of ventilator-related pneumonia. This study was a retrospective cohort study. This study was conducted from October 2017 to February 2018 and collected data from the intensive care unit of a medical center in southern Taiwan from January 2013 to May 2016. The control group (enrolled from January 2013 to June 2014) received oral hygiene. The experimental group (enrolled from July 2014 to December 2015) received five-item ventilator-associated pneumonia prevention care bundles, which consisted of (1) elevation of the head of the bed to 30–45°; (2) daily oral care with 0.12−0.2% chlorhexidine twice daily; (3) daily assessment of readiness to extubate; (4) daily sedative interruption; and (5) emptying water from the respirator tube. Results showed the incidence of ventilator-associated pneumonia in the bundle group was significantly less than the oral hygiene group (*p* = 0.029). The factors that significantly affected the incidence of ventilator-associated pneumonia were ventilator-associated pneumonia care bundle, ventilator-days, and intensive care unit length of stay. A significant reduction in ventilator-associated pneumonia rate in the bundle group compared to the oral hygiene group (OR = 0.366, 95% CI = 0.159–0.840) was observed, with 63.4% effectiveness. Application of an automatic medical information system to implement bundle care can significantly reduce the incidence of ventilator-associated pneumonia.

## 1. Introduction

Ventilator-associated pneumonia is a common hospital acquired infection in patients in the intensive care unit [1,2]. The incidence of ventilator-associated pneumonia in patients with respirators in the intensive care unit is as high as 21–42%, and the mortality rate is 8–46%; it also causes patients to stay longer in the hospital and increases medical costs [3]. In order to reduce the incidence of ventilator-associated pneumonia in the hospital and promote the quality of medical care, hospitals implement related care measures to prevent ventilator-associated pneumonia. Studies have shown that oral care with chlorhexidine can effectively reduce the incidence of ventilator-associated pneumonia in patients [1,4,5]. Researchers suggested that using chlorhexidine mouthwash for 30 s can effectively reduce the incidence of ventilator-associated pneumonia [1]. Oral care with chlorhexidine was found by some researchers to have no significant effect on reducing mortality, mechanical ventilation duration, intensive care unit length of stay, or total number of days spent in the hospital [1,6]. In order to improve the preventive effect of ventilator-associated pneumonia, various organizations have proposed ventilator-associated pneumonia prevention care bundles [7,8], and various intensive care units have implemented ventilator-associated pneumonia prevention bundles [9,10,11,12]. Research suggests that ventilator-associated pneumonia care bundle can significantly reduce the incidence of ventilator-associated pneumonia, but various studies have different contents in the care bundles and have inconsistent effects on reducing mechanical ventilation duration and mortality rate [10,11,12]. The research of Al-Mousa et al. (2018) [9] and Goel et al. (2019) [10] explored the effects of five-element ventilation care bundles, consisting of: (1) elevation of the head of the bed at 30–45°; (2) daily sedative interruption and daily assessment of readiness to extubate; (3) peptic ulcer disease prophylaxis; (4) deep venous thrombosis prophylaxis; and (5) daily oral care with chlorhexidine. Khan et al. (2016) [11] studied the implementation of a seven-element care bundle, including head-of-bed elevation 30–45, daily sedation vacation and assessment for extubation, peptic ulcer disease prophylaxis, deep vein thrombosis prophylaxis, oral care with chlorhexidine, endotracheal intubation with in-line suction and subglottic suctioning, and maintenance of endotracheal tube cuff pressure at 20–30 mmHg.

Ochoa-Hein et al. (2019) [12] studied the effects of the ventilator-associated pneumonia preventive bundle, including hand hygiene, tooth brushing, oral chlorhexidine gluconate, head of bed elevation >30°, patient position changes, and standardization of the protocol for early weaning from endotracheal tubes. Few studies have explored the effects of the five-element care bundle content in this study. The main purpose of this study was to prevent the occurrence of ventilator-associated pneumonia. For the oral hygiene group in the current study, the method of mouth care was to brush patients’ teeth with toothpaste and mouth care with chlorhexidine for 30–60 s from January 2013 to June 2014, but the incidence of ventilator-associated pneumonia was not significantly reduced. Therefore, from July 2014 to May 2016, five-element ventilator-associated pneumonia care bundles promoted by Taiwan Centers for Disease Control [13] were used to reduce the incidence of ventilator-associated pneumonia. The five-element ventilator-associated pneumonia care bundles promoted by the Taiwan Centers for Disease Control (2017) include: (1) elevation of the head of the bed to 30–45°; (2) oral care with 0.12–0.2% chlorhexidine mouthwash twice daily; (3) daily assessment of readiness to extubate; (4) daily sedative interruption; and (5) empty water from the respirator tube [13]. This research team used the automatic medical information system to achieve the implementation of five-element measures to prevent ventilator-associated pneumonia. It was the information-based automatic screening reminder system. This study compared the occurrence rates of ventilator-associated pneumonia in the adult intensive care unit patients before and after implementation of ventilator-associated pneumonia care bundle (oral hygiene group enrolled from January 2013 to June 2014 versus bundle group enrolled from July 2014 to December 2015). Further, this study explored the related factors that affect the occurrence of ventilator-associated pneumonia.

## 2. Methods

### 2.1. Study Design

This study was a retrospective cohort study. It was conducted from October 2017 to February 2018 and collected data from the intensive care unit of a medical center in southern Taiwan from January 2013 to May 2016. The purpose of this study was to conduct pre-intervention and post-intervention tests to explore the effects of ventilator-associated pneumonia care bundle intervention.

### 2.2. Setting and Subjects

This study was conducted in the adult intensive care unit of a medical center. All adult patients with mechanical ventilators admitted to the adult intensive care unit between 1 January 2013 and 31 December 2015, were enrolled in this study. The exclusion criteria were below 20 years of age, and patients who had been diagnosed with pneumonia before mechanical ventilation placement.

### 2.3. Data Sources

Data were collected from the patients’ medical records. Data collection included patient age, gender, number of days on a ventilator, length of stay in the intensive care unit, total number of days admitted to the hospital, diagnosis, past disease history, and whether ventilator-associated pneumonia had occurred. The definition of respirator-associated pneumonia in Taiwan is implemented in accordance with the guidelines set by the National Nosocomial Infection Surveillance [14]. The list of patients with ventilator-associated pneumonia was provided by the hospital infection control department for this study.

### 2.4. Intervention

The intervention measures used to prevent ventilator-associated pneumonia in ventilator patients in this study were divided into two groups. One was the oral hygiene group (enrolled in intensive care January 2013 to June 2014). The other was prevention ventilator-associated pneumonia care bundle group (enrolled in intensive care July 2014 to December 2015).

### 2.5. Oral Hygiene Group

For this group, only oral care was performed, including use of toothbrush with toothpaste, and mouth care with chlorhexidine for 30–60 s twice a day. 

### 2.6. Prevention Ventilator-Associated Pneumonia Care Bundle Group

The group implemented five-item ventilator-associated pneumonia prevention care bundles, including (1) elevation of the head of the bed to 30–45°; (2) daily oral care with 0.12–0.2% chlorhexidine twice daily; (3) daily assessment of readiness to extubate; (4) daily sedative interruption; (5) emptying water from the respirator tube. This medical team innovated and developed automatic medical information system to ensure that the five-element measures to prevent ventilator-associated pneumonia were being implemented. The automatic medical information system had three main features: (1) five-element ventilator-associated pneumonia care bundle checklist: This medical team formulated a five-element ventilator-associated pneumonia care bundle checklist in automatic medical information system, and the nurse checked and recorded the results in this system every day. (2) The automatic medical information system automatically judged and reminded medical personnel when the respirator can stop being used: The medical team set the conditions for discontinuing respirator use in the automatic medical information system, including no fever within 24 h, PaO_2_/FiO_2_ > 200 mmHg, FiO_2_ < 45%, and PEEP ≤ 5 cm H2O. The automatic medical information system automatically judged the parameter conditions for the past 24 h at 7 o’clock every morning. When it was judged that the conditions for extubation were met, the system automatically displayed a reminder of “Please evaluate extubation”. Through the judgment and reminder of automatic medical information system, medical staff could allow patients to enter the process of preparing for extubation as soon as possible. (3) Established the standard procedure of oral care with 0.12–0.2% chlorhexidine for patients with indwelling endotracheal tube in the automatic medical information system, and provided staff with the procedures to perform oral care of patients.

### 2.7. Data Analysis

Descriptive and inferential statistical methods were used. Mean values and standard deviations were presented for normally distributed continuous variables, median values and quartiles (Q1, Q3) for skewed continuous variables, while frequencies and percentages were used for categorical variables. We conducted a difference analysis on the personal characteristics of the two groups (oral hygiene group and care bundle group), using Chi-square test for categorical variables and *t*-test or Mann–Whitney test for continuous variables. We verified that the data conformed to the assumptions and preconditions of the methods used to analyze them. The results showed that the age was normally distributed, but the ventilator days, intensive care unit days, and hospitalization days were not normally distributed. Therefore, Mann–Whitney U-test was used to evaluate the statistical significance between the two groups for ventilator days, intensive care unit days, and hospitalization days. Chi-square test was used for the two groups (oral hygiene group and care bundle group) and for the occurrence of ventilator-associated pneumonia to explore the influence of different interventions in the two groups on the occurrence of VAP. Next, we used Chi-square test or *t*-test or Mann–Whitney U test to explore the influencing factors of ventilator-associated pneumonia. Finally, logistic regression was used to predict the influencing factors and occurrence probability of ventilator-associated pneumonia. The occurrence of ventilator-associated pneumonia was a dependent variable. All related factors affecting the occurrence of ventilator-associated pneumonia, including group, ventilator days, intensive care unit days, hospitalization days, and past disease history, were included as independent variables. *p*-values < 0.05 were considered statistically significant. The data were managed in Excel (Microsoft Corp, Redmond, Washington) and analyzed using IBM SPSS Statistics for Windows, version 20.0 (IBM Corp., Armonk, NY, USA). 

### 2.8. Ethics Considerations

This study was reviewed and approved by the Human Research Ethics Review Committee of the Medical Center (VGHKS16-CT10-02).

## 3. Results

### 3.1. Patient Characteristics

A total of 1475 patients participated in this study. There were 779 in the oral hygiene group and 696 in the ventilator-associated pneumonia prevention bundles group. The basic information of the participants was shown in Table 1. The average age of the patients was 66.5 ± 14.0 years old. The majority were males (67.4%). Most of them had a past medical history (89.5%). The differences of personal characteristics between oral hygiene group and ventilator-associated pneumonia care bundle group were tested, and the results were significantly different only for the presence or absence of past medical history. The ventilator-associated pneumonia care bundle group had more past medical history than oral hygiene group. The other variables were not significantly different between the two groups. Overall, the basic data of the two groups of patients were homogeneous. In the subsequent analysis of related factors affecting the occurrence of ventilator-associated pneumonia, past medical history was used as a covariate for control.

### 3.2. The Incidence of Ventilator-Associated Pneumonia

As shown in Table 2, a total of 32 people (2.2%) had ventilator-associated pneumonia, with 23 cases in the oral hygiene group and nine in the care bundle group. The χ^2^-*test* results showed a significant difference (χ^2^ = 4.769, *p* = 0.029), indicating the occurrence of ventilator-associated pneumonia in the care bundle group was significantly less than oral hygiene group.

### 3.3. The Difference between Ventilator-Associated Pneumonia Cases and Non-Ventilator-Associated Pneumonia Cases

As shown in Table 3, the results showed that the cases with ventilator-associated pneumonia and the cases without ventilator-associated pneumonia were significant different in the variables of ventilator days, average intensive care unit length of stay, average hospital day, and groups (*p* < 0.05). It showed the ventilator days, intensive care unit length of stay, and hospital day with ventilator-associated pneumonia cases were significantly higher than those without ventilator-associated pneumonia. There were significantly more ventilator-associated pneumonia cases in the oral hygiene group than in the care bundle group.

### 3.4. Logistic Regression for Predicting Ventilator-Associated Pneumonia

As shown in Table 4, the factors that affect the occurrence of ventilator-associated pneumonia are discussed using logistic regression, stepwise estimation, and all relevant factors are put into covariance to exclude errors that may affect the accuracy of the experiment. The covariance included was groups, intensive care unit length of stay, ventilator days, hospital days, past disease history. As a result, the best model for regression, the group (OR = 0.366, 95% CI = 0.159–0.840), Ventilator days (OR = 1.031, 95% CI = 1.010–1.052), intensive care unit days (OR = 1.029, 95% CI = 1.004–1.055), three variables significantly affect the occurrence of ventilator-associated pneumonia. The odds ratio (OR) of the ventilator-associated pneumonia occurrence in the bundle group was 0.366 times that of the oral hygiene group, showing that the bundle group had a 63.4% reduction in the OR of ventilator-associated pneumonia occurrence. For each additional day of ventilator usage, the OR of ventilator-associated pneumonia occurrence is 1.031 times; for each additional day of intensive care unit stay time, the OR of ventilator-associated pneumonia occurrence is 1.029 times the original.

## 4. Discussion

After the implementation of the five-item ventilator-associated pneumonia prevention care bundles, this study found a significantly lower incidence of ventilator-associated pneumonia than for the oral hygiene group, which is consistent with the results of other studies showing that the implementation of ventilator-associated pneumonia care bundles can significantly reduce the incidence of ventilator-associated pneumonia [3,9,10]. Collectively, current and past studies show that although the contents of ventilator-associated pneumonia care bundles implemented may be different; they all have had the effect of reducing the occurrence of ventilator-associated pneumonia. This consistency in the literature is dependent on the consensus among medical team members and the assurance of bundle compliance, which are keys to ensuring the effectiveness of the care bundle [10,11]. However, the implementation of the ventilator-associated pneumonia care bundle in this study showed no significant difference in reducing number of days admitted to hospital or intensive care unit length-of-stay, which is consistent with the results of Khan et al. (2016) [11]. Those researchers showed that the implementation of bundle care did not significantly reduce the mechanical ventilation duration nor intensive care unit length of stay for patients. However, the results are inconsistent with Ochoa-Hein et al. (2019) [12]. That study showed that the implementation of ventilator-associated pneumonia care bundle significantly reduced mechanical ventilation duration days of patients. Further research is necessary. This study used logistic regression; it was found that the factors that significantly affected the occurrence of ventilator-associated pneumonia were the presence or absence of a care bundle, number of days on a ventilator, and number of days in intensive care unit. This result is consistent with the results of Al-Mousa et al. (2018) [9], which showed that after controlling for mechanical ventilation duration, hospital days, and intensive care unit stay time, the risk of ventilator-associated pneumonia occurrence in patients who received the care bundle was significantly reduced, and mechanical ventilation duration and intensive care unit stay time significantly increased the risk of ventilator-associated pneumonia occurrence.

The results of this study showed that the OR of ventilator-associated pneumonia occurrence of bundles group was 0.366 times that of the oral hygiene group, reducing the OR of ventilator-associated pneumonia occurrence by 63.4%. For each additional ventilator-day, the OR of ventilator-associated pneumonia occurrence is 1.031 times, which is consistent with the results of the literature [9]. It was shown that as Ventilator days increased, so did the chance of ventilator-associated pneumonia, so it is very important to remove the endotracheal tube as early as possible to reduce the ventilator days. For each additional day of intensive care unit stay time, the OR of ventilator-associated pneumonia occurrence is 1.029 times, indicating that the longer the intensive care unit stay time, the higher the chance of ventilator-associated pneumonia occurrence. Therefore, the medical team needs mutual cooperation to improve the results of patient outcomes and early transfer out of the intensive care unit.

This study was limited to results that were conducted only at a medical center and it only found results of the implementation of a five-item ventilator-associated pneumonia care bundle. To continuously improve the quality of care and promote better care results. In the future, it is recommended to explore the impact of reducing the number of ventilator days and intensive care unit stays on reducing the occurrence of ventilator-associated pneumonia, as well as the impact of other types of care bundles. This was a retrospective study, and it is necessary to conduct a better randomized controlled trial design to improve the reliability and validity of the research results.

## 5. Conclusions

This study suggested that a five-element preventative ventilator-associated pneumonia care bundle can significantly reduce the incidence of ventilator-associated pneumonia. The significant factors affecting the occurrence of ventilator-associated pneumonia are the presence or absence of care bundles, ventilator days, and intensive care unit days. It is recommended that ventilator-associated pneumonia care bundles should be used, and further study is needed. Therefore, medical teams need to cooperate with each other. If the automated medical information system can provide busy medical staff with more accurate data to help patients in time to reduce the chance of ventilator-associated pneumonia.

### Relevance to Clinical Practice

It is recommended to establish a complete simplified and automated reminder mechanism using technology and modern information systems to improve the quality of care. It is recommended to conduct a more comprehensive discussion to reduce the impact of ventilator days and length of stay in the intensive care unit on the incidence of ventilator-associated pneumonia, as well as the impact of different types of care bundles on reducing the incidence of ventilator-associated pneumonia to provide better quality of care and more good results.

## Figures and Tables

**Table 1:** Personal Characteristics of Study Participants (*N* = 1475).

Variables		Total (*N* = 1475) Mean ± SD/*n* (%)	Oral Hygiene (*n* = 779) Mean ± SD/*n* (%)	Care Bundle (*n* = 696) Mean ± SD/*n* (%)	χ^2^/*t*/Z	*p*
Age (Years)		66.5 ± 14.0	66.8 ± 14.1	66.2 ± 14.0	0.744	0.457 ^a^
Gender					1.793	0.181 ^b^
	Male	994 (67.4)	537 (68.9)	457 (65.7)		
	Female	481 (32.6)	242 (31.1)	239 (34.3)		
Diagnosis					9.277	0.233 ^b^
	Cardiovascular	959 (65.0)	522 (67.0)	437 (62.8)		
	Lung disease	186 (12.6)	97 (12.5)	89 (12.8)		
	Kidney disease	46 (3.1)	23 (3.0)	23 (3.3)		
	Infectious	123 (8.3)	60 (7.7)	63 (9.1)		
	Gastrointestinal	45 (3.1)	22 (2.8)	23 (3.3)		
	Brain disease	35 (2.4)	21 (2.7)	14 (2.0)		
	Cancer	30 (2.0)	16 (2.1)	14 (2.0)		
	Others	51 (3.5)	18 (2.3)	33 (4.7)		
Past disease history					5.783	0.016 ^b^
	Yes	1320 (89.5)	683 (87.7)	637 (91.5)		
	No	155 (10.5)	96 (12.3)	59 (8.5)		
Hypertension					2.005	0.157 ^b^
	Yes	866 (58.7)	444 (57.0)	422 (60.6)		
	No	609 (41.3)	335 (43.0)	274 (39.4)		
Diabetes					0.881	0.348 ^b^
	Yes	539 (36.5)	276 (35.4)	263 (37.8)		
	No	936 (63.5)	503 (64.6)	433 (62.2)		
Cardiovascular disease					0.941	0.332 ^b^
	Yes	648 (43.9)	333 (42.7)	315 (45.3)		
	No	827 (56.1)	446 (57.3)	381 (54.7)		
Lung disease					1.947	0.163 ^b^
	Yes	83 (5.6)	50 (6.4)	33 (4.7)		
	No	1392 (94.4)	729 (93.6)	663 (95.3)		
Infectious disease					0.244	0.621 ^b^
	Yes	29 (2.0)	14 (1.8)	15 (2.2)		
	No	1446 (98.0)	765 (98.2)	681 (97.8)		
Ventilator-days		8.1 ± 13.3	7.9 ± 12.1	8.3 ± 14.5	−0.551	0.581 ^c^
Median (Q1, Q3)		2.8 (1.0, 8.9)	2.9 (1.0, 9.2)	2.8 (1.0, 8.9)		
Intensive care unit days		9.2 ± 10.7	9.3 ± 10.7	9.0 ± 10.7	0.508	0.611 ^c^
Median (Q1, Q3)		5.2 (3.0, 11.8)	5.2 (3.0, 11.9)	5.2 (3.0, 11.7)		
Hospital days		27.7 ± 22.5	27.0 ± 20.8	28.4 ± 24.2	−0.753	0.452 ^c^
Median (Q1, Q3)		20.9 (13.9, 35.9)	20.9 (13.8, 36.8)	21.2 (13.9, 35.0)		

SD, standard deviation; Q1, lower quartiles; Q3, upper quartiles; ^a^, *t*-test; ^b^, χ^2^-test; ^c^, Mann–Whitney U-test.

**Table 2:** The Incidence of Ventilator-Associated Pneumonia (*N* = 1475).

Variables	Total (*N* = 1475) *n* (%)	Oral hygiene (*n* = 779) *n* (%)	Care bundle (*n* = 696) *n* (%)	χ^2^	*p*
Ventilator-associated Pneumonia				4.769	0.029
Yes	32 (2.2)	23 (3.0)	9 (1.3)		
No	1443 (97.8)	756 (97.0)	687 98.7)		

SD, standard deviation.

**Table 3:** Ventilator-Associated Pneumonia Related Factors (*N* = 1475).

Variables		Ventilator Associated Pneumonia Cases (*n* = 32) Mean ± SD/*n* (%)	Non-Ventilator Associated Pneumonia Cases (*n* = 1443) Mean ± SD/*n* (%)	χ/*t*/Z	*p*
Age (years)		67.1 ± 15.3	66.5 ± 14.0	0.250	0.803 ^a^
Gender				0.299	0.584 ^b^
	Male	23 (71.9)	971 (67.3)		
	Female	9 (28.1)	472 (32.7)		
Diagnosis				6.657	0.465 ^b^
	Cardiovascular	19 (59.4)	940 (65.1)		
	Lung disease	8 (25.0)	178 (12.3)		
	Kidney disease	0 (0.0)	46 (3.2)		
	Infectious	3 (9.4)	120 (8.3)		
	Gastrointestinal	1 (3.1)	44 (3.0)		
	Brain disease	0 (0.0)	35 (2.4)		
	Cancer	0 (0.0)	30 (2.1)		
	Others	1 (3.1)	50 (3.5)		
Past disease history				0.138	0.710 ^b^
	Yes	28 (87.5)	1292 (89.5)		
	No	4 (12.5)	151 (10.5)		
Ventilator days		26.8 ± 22.9	7.6 ± 12.7	7.047	0.000 ^c^
Median (Q1, Q3)		23.5 (10.6, 30.7)	2.8 (1.0, 8.6)		
Intensive care unit days		24.4 ± 15.9	8.9 ± 10.3	6.600	0.000 ^c^
Median (Q1, Q3)		22.4 (14.7, 30.5)	5.2 (3.0, 11.1)		
Hospital days		39.3 ± 29.1	27.4 ± 22.3	3.100	0.002 ^c^
Median (Q1, Q3)		33.2 (20.4, 42.5)	20.9 (13.8, 35.0)		
Group				4.769	0.029 ^b^
	Oral hygiene	23 (71.9)	756 (52.4)		
	care bundle	9 (28.1)	687 (47.6)		

SD, standard deviation; Q1, lower quartiles; Q3, upper quartiles; ^a^, *t*-test; ^b^, χ^2^-test; ^c^, Mann–Whitney U-test.

**Table 4:** The Logistic Regression Model for Predicting Ventilator-Associated Pneumonia.

Variables	Coeff (SE)	OR (95% CI)	*p*-Value
Groups (care bundle-oral hygiene)	−1.01 (0.42)	0.367 (0.16–0.84)	0.018
Ventilator days	0.03 (0.01)	1.03 (1.01–1.05)	0.003
Intensive care unit days	0.03 (0.01)	1.03 (1.00–1.06)	0.024
Constant	−4.29 (0.28)	0.01	0.000

Logistic regression model including the three variables (groups, ventilator days, intensive care unit days) in the table. Coeff, coefficient of the logistic regression; SE, standard error; OR, odds ratio; CI, confidence interval. Oral hygiene was the reference group for Groups.

## Data Availability

The research data was provided from the data database of Kaohsiung Veterans General Hospital.
